# The calculated voyage: benchmarking optimal strategies and consumptions in the Japanese eel’s spawning migration

**DOI:** 10.1038/s41598-024-74979-0

**Published:** 2024-10-31

**Authors:** Gen Li, Yu-Lin Chang, Yasumasa Miyazawa, Ulrike K. Müller

**Affiliations:** 1 Center for Mathematical Science and Advanced Technology, Japan Agency for Marine-Earth Science and Technology, Yokohama, Japan; 2 Application Laboratory, Japan Agency for Marine-Earth Science and Technology, Yokohama, Japan; 3 Department of Biology, California State University, Fresno, USA

**Keywords:** Migration, Eel, Optimization, Energy consumption, Swimming speed and depth, Ocean current, Animal migration, Fisheries, Computational models

## Abstract

Eels migrate along largely unknown routes to their spawning ground. By coupling Zermelo’s navigation solution and data from the Japan Coastal Ocean Predictability Experiment 2 (JCOPE2M), we simulated a range of seasonal scenarios, swimming speeds, and swimming depths to predict paths that minimize migration duration and energy cost. Our simulations predict a trade-off between migration duration and energy cost. Given that eels do not refuel during their migration, our simulations suggest eels should travel at speeds of 0.4–0.6 body-length per second to retain enough energy reserves for reproduction. For real eels without full information of the ocean currents, they cannot optimize their migration in strong surface currents, thus when swimming at slow swimming speeds, they should swim at depths of 200 m or greater. Eels swimming near the surface are also influenced by seasonal factors, however, migrating at greater depths mitigates these effects. While greater depths present more favorable flow conditions, water temperature may become increasingly unfavorable, dropping near or below 5 °C. Our results serve as a benchmark, demonstrating the complex interplay between swimming speed, depth, seasonal factors, migration time, and energy consumption, to comprehend the migratory behaviors of Japanese eels and other migratory fish.

## Introduction

Long-distance migratory behaviors occur in many animal taxa^1–4^. Animals often migrate along well-established routes, yet what determines those routes is still being researched^5–9^. Past field and modeling work has shown that long-distance migratory routes are often co-determined by time- and energy-constraints, as animals minimize migration time, maximize energy efficiency, and divide their time and energy between refueling and traveling^6,10–16^.

Migratory animals must balance the time lost to refueling against the energy cost of carrying fuel, which determines how far and how long animals go between refueling sites. At one extreme, the distance between refueling sites is effectively zero as some ungulates migrate following or anticipating seasonal changes in resource availability (green wave hypothesis)^17–19^. At the other extreme, eels complete their migration without refueling. Migratory speed, defined as the overall displace rate during migration, includes the time for acquiring energy as well as for transport^10^, and should not be confused with pure transport (swimming, flying or walking) speed^10^. Refueling reduces migratory speed: the migratory speeds of birds and ungulates are typically eight times lower than their respective pure flying and walking speeds^10,19^. Swimming migrants operate in a medium that is much denser than air and has a density similar to their own. As a result, they face the least additional energy expenditure related to overcoming their weight, incurring the lowest cost from carrying extra weight. However, at the same speed, they experience greater fluid resistance, which constrains their migratory speeds^10,20^.

Migratory routes also reflect animals’ need to navigate around ecological and natural barriers^21–25^, including winds and ocean currents. Unlike the effects of wind on bird migration^22,26,27^, we know little about the effect of ocean currents on migrating fish^27–30^. Ocean currents, such as the North Atlantic^31^ and Pacific Equatorial currents^32^, typically move at speeds that are within the same order of magnitude (10^–1^ m/s) as the cruising speeds of adult fish and late-stage larvae^33^, including the commercially important European eels^34^ and Atlantic salmon^35^.

Anguillid eels are globally distributed migratory fish that return to specific spawning areas, migrating thousands of miles^36^. Extensive studies over recent decades have shed light on some details about the Japanese eel *Anguilla japonica*^37,38^. Japanese eels predominantly inhabit freshwater environments surrounding the western Pacific Ocean and spawn along the western flank of the West Mariana Ridge^39^. At the end of the growth yellow eel phase, eels undergo a second metamorphosis called ‘silvering’. The silver eels start their spawning migration from continent growth habitats toward spawning area^40^. The spawning season commences in April and culminates in August^41^. The Japanese eel’s spawning area resides within the North Equatorial Current’s (NEC) continuous westward flow, allowing larval eels to drift with the current before following the Kuroshio Current to their freshwater habitats. The speed of ocean currents exceeds the swimming capacity of larval eels, so their migration route is dictated by ocean currents’ direction^41^. Whereas eel larvae benefit from migrating along the prevailing ocean currents, adults face travelling against those same currents when they return to their spawning grounds. Experimental studies indicate that silver eels have sufficient fat reserves for their long-distance migration journey (studies on European eel^42,43^).

Numerical simulations, leveraging ocean current data and particle-based methods, have been used to explore the eel larvae’s migration route, duration, and success rate^44–47^. Yet the migratory routes and navigation methods used by silver eels remain unidentified^48^. While field surveys are crucial to resolving this mystery, theoretical predictions from numerical models offer valuable insights. First, predictive models can be used to calculate likely migration routes, and these predictions can help target field surveys. Second, mechanistic models can be used to develop performance benchmarks^30^ and examine the relation between animals’ characteristics (such as behavioral choices or energy needs), environmental factors (such as ecological barriers), and performance outcomes (such as migration time). Combining such mechanistic models with experimental and observational study can help gain a better understanding of causal relations, such as the factors that co-determine migration routes^14^. In this study, we use numerical models to predict the optimal path from arbitrary starting points to a predefined spawning area through a system of known ocean currents, including known seasonal and geographic changes in those currents. Zermelo was the first to conceptualize and provide a general solution to build such a path-optimization model by employing optimal control theory to create a partial differential equation, referred to as Zermelo’s solution^49,50^ and his model has been used successfully to study animal migration^14,15,30^. This solution simplifies the issue of determining optimal headings at every potential point in space and time to an initial-value problem^30,50,51^, which can be solved numerically. The model makes several simplifying assumptions. First, it assumes that the swimmer’s migratory speed is constant, which is a justifiable approximation for eels given their unique migratory strategy: variations in a swimmer’s migratory speed are mainly associated with the switching between travelling and refueling^10^, yet eels cease feeding during the silver-eel stage and complete their long-distance migrations without refueling^43,52–54^. Hence eels’ pure swimming speed closely approximates their migratory speed^55^. Second, the model assumes purely horizontal movement. This assumption is not a valid approximation for many aquatic migratory species, including eels, which perform daily vertical migrations^34,56–58^. While diel vertical migrations add little to the overall migration distance and time, they cause eels to traverse across changing current and temperature regimes^57,59^. This diel variation in flow does not meet the conditions required for the Zermelo’s solution, which is based on a steady flow field. To accommodate vertical migrations in our study, we model migration at a range of swimming depths observed in Japanese eels and examine the effect of depth on optimal migration path and migration time. Third, the model assumes that the migrant has ‘full knowledge’ of the ocean flows through which it migrates, a valid approximation for innate migration routes, migration routes learned through natal homing, and marine larval dispersal, which reflect and sometimes exploit prevailing wind and current patterns^4,15,60–64^. This last assumption makes this model a valuable heuristic tool for assessing whether migrants optimize migration routes by comparing observed with predicted routes, as shown in previous studies^14,15^. Another way of looking at the ‘full knowledge’ condition is that this assumption implies that ocean currents are stable across time and hence predictable. Zermelo’s solution is a deterministic model that does not take into account the probability of the encountered ocean currents. It therefore predicts an optimal migration route based on prevailing currents, but it does not predict the likelihood of that path being optimal. Although stochastic models have been developed to study migration^13^, deterministic models remain attractive because they are computationally efficient, straightforward to implement, and more transparent, facilitating mechanistic interpretations of the predicted migration routes^14,15^. To assess the role of variable flow conditions, this study quantifies the spatial and temporal variability of the ocean currents to assess their influence on predicted migration routes.

In this study, we aim to predict optimal migration routes based on minimal time and energy requirements for Japanese eel migration. To achieve this, we employ a modified Zermelo algorithm in a spherical coordinate system^30,51^, coupled with our high-resolution ocean current database for the western North Pacific Ocean^65^.

## Results

The optimal migration paths of the eel were predicted using Zermelo’s approach^50^ in a reverse-time system (see “Numerical strategy” section), coupled with a West Pacific Ocean flow database sourced from the Japan Coastal Ocean Predictability Experiment 2’s (JCOPE2) ocean circulation forecast system^65^. We performed multiple simulations for a range of swimming speeds and depths observed in eels to map the possible migration paths. We simulated the effects of season (winter versus summer), migration depth and its effect on water temperature and ocean currents.

### Optimal migration performance with respect to swimming speed and depth

**Fig. 1:**
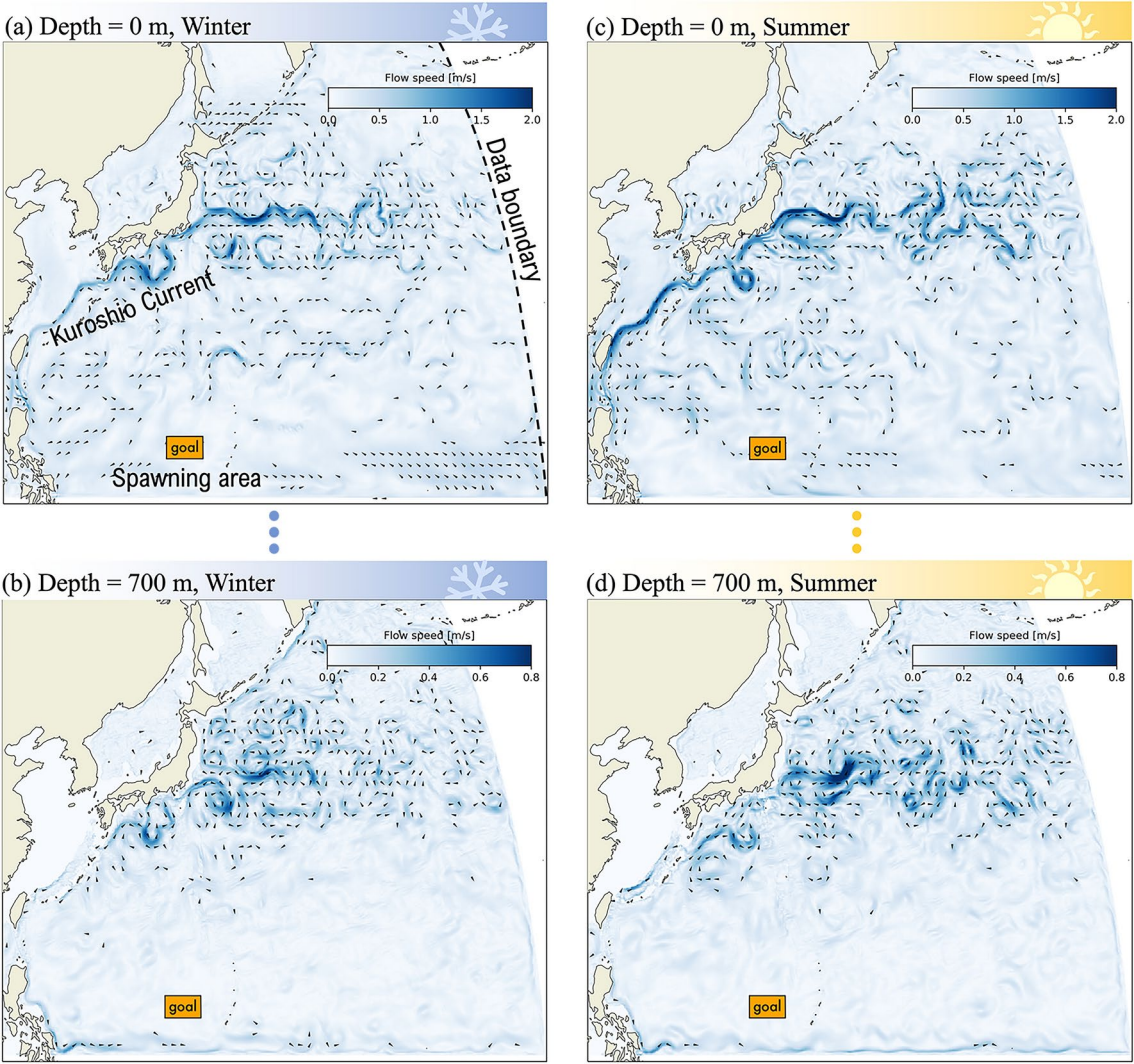
Comparison of Oceanic Flow Fields for Winter and Summer Scenarios at the shallowest and deepest depths used in this study. (**a**) Surface-level (0 m) flow field in the Winter Scenario; (**b**) Deep-sea (700 m) flow field in the Winter Scenario; (**c**) Surface-level (0 m) flow field in the Summer Scenario; (**d**) Deep-sea (700 m) flow field in the Summer Scenario. The Winter Scenario flow field is derived from the average monthly flow for January 2020, while the Summer Scenario is based on the average monthly flow for July 2020. The spawning area is indicated by ‘goal’. Please note that the color scale bars differ between the panels.

For the winter-scenario, we averaged flow in time over the month of January 2020. We also averaged in space in 100-m depth steps from the surface down to 700 m. Flow speed is highest at the surface and drops conspicuously with depth, as evident in the difference in scale bars between Fig. 1a and b. Prominent currents, such as the Kuroshio Current (KC) and the North Equatorial Current (NEC) are stronger and most distinct near the surface (Fig. 1a).

**Fig. 2:**
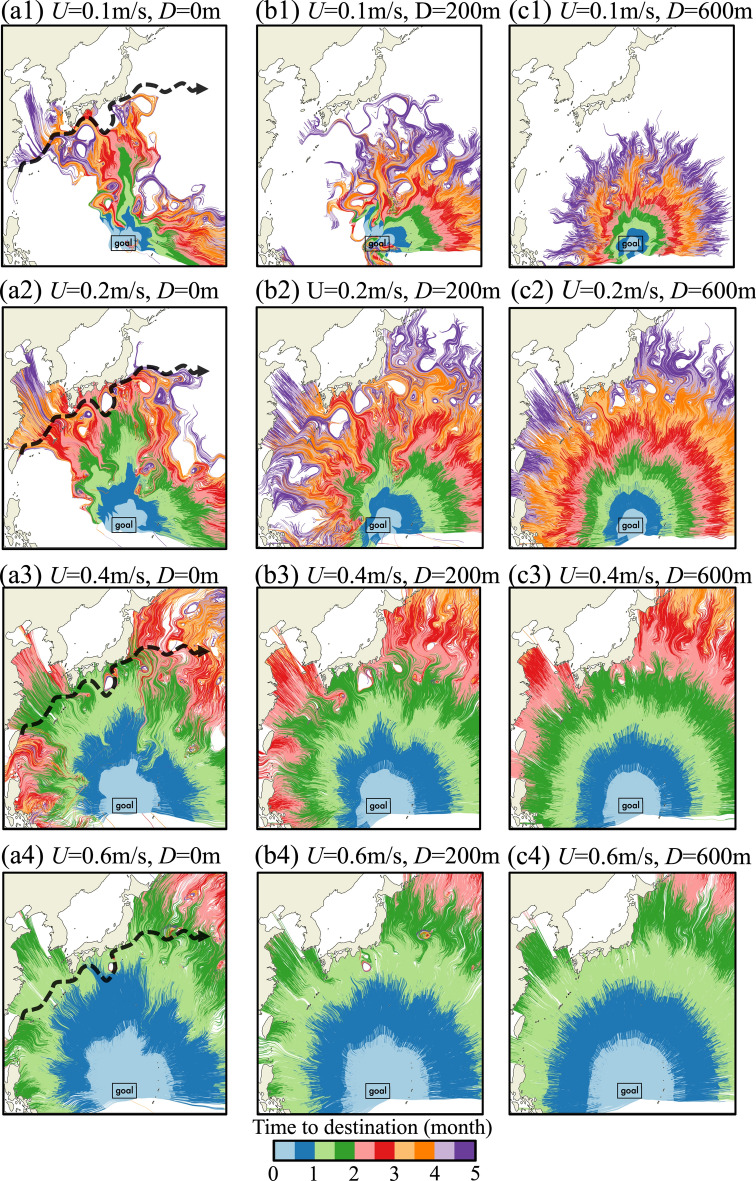
Optimal migration path in the Winter Scenario at Various Depths. Panels (**a1**–**a4**) depict optimal migration paths at the surface (Depth = 0 m); panels (**b1**–**b4**) illustrate optimal migration paths at a moderate depth (Depth = 200 m); panels (**c1**–**c4**) present optimal migration paths in the deep sea (Depth = 600 m). The color along the optimal migration path indicates the migration time from the arbitrary start point to the spawning area (indicated by ‘goal’). At *D* = 0, the position of the Kuroshio current is marked with a black dashed line to visually represent the interaction between the migratory paths and the ocean currents.

**Fig. 3:**
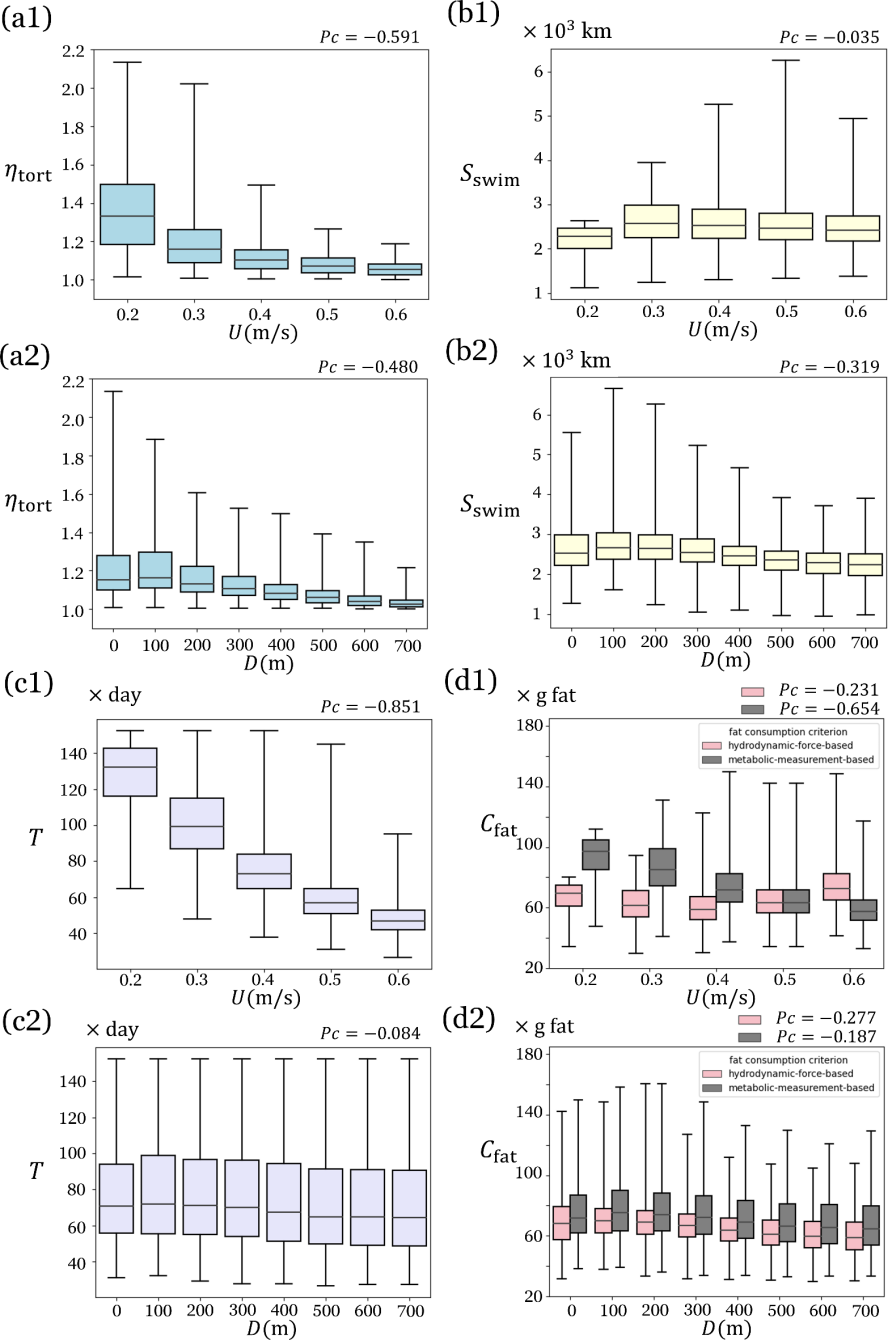
Statistical analysis across all established optimal migration paths to major habitats (Fig. S1, Supplementary Information) in the Winter Scenario. (**a1**) The variation of the Tortuosity Index ${\eta }_{\text{tort}}$ with respect to swimming speed *U*, indicating that higher speeds result in straighter optimal migration paths. (**a2**) The variation of ${\eta }_{\text{tort}}$ with respect to swimming depth *D*, showing that greater depths lead to straighter optimal migration paths. (**b1**) The variation of Swimming Distance ${S}_{\text{swim}}$ with respect to swimming speed, indicating that higher speeds may slightly reduce swimming distance. (**b2**) The variation of ${S}_{\text{swim}}$ with respect to time swimming depth, showing that greater depths lead to less swimming distance. (**c1**) The variation of optimal migration time with respect to swimming speed, indicating that higher speed significantly reduces migration time. (**c2**) The variation of optimal migration time with respect to swimming depth. (**d1**) The variation of fat consumption with respect to swimming speed. (**d2**) The variation of fat consumption with respect to swimming depth. In (**d1**) and (**d2**), two groups of results based on different fat consuming criterions are displayed, where “hydrodynamic measurement” group represents the results using Eq. (2), and “metabolic measurement” group represents the results using Eq. (3). For each panel, Pearson correlation coefficient (*Pc*) is displayed at upper-right corner. Linear regression analyses on all the data in Fig. 3 are provided in Supplementary Information, §S5.

We determined the optimal migration paths at eight depth intervals, every 100 m from 0 to 700 m, for six swimming speeds between 0.1 and 0.6 m/s, resulting in 48 simulation scenarios (representative scenarios in Fig. 2, complete data set in Supplementary Fig. 1), using value ranges conforming with field and laboratory observations^66–69^. We found that swimming speed has a profound effect on optimal migration trajectories at low swimming depths. A comparison between the two extremes—swimming slowly near the surface (Fig. 2 (a1)) versus swimming fast at 600 m depth (Fig. 2 (c4))—shows that swimming fast through weak currents results in optimal migration paths that form an almost perfectly radial pattern with its center at the spawning ground, indicating that trajectories are nearly straight and time to destination is effectively proportional to the distance travelled (The overall statistics are provided later in Fig. 3, and detailed regional statistics are presented in Fig. S2 in the Supplementary Information). In contrast, eels swimming slowly near the surface follow contorted trajectories shaped by local currents and eddies.

Swimming speed and depth greatly affect which starting locations allow eels to reach their spawning grounds. Swimming slowly near the surface severely restricts available starting locations (Fig. 2 (a1) and (a2)). Eels cannot reach the spawning grounds from the west due to the westward NEC, evident in a vacant area of optimal paths to the west of the spawning area. Although our model predicts possible starting locations to the east of the spawning ground, there are no known eel habitats to the east from which eels might initiate migration. Eels living to the north of the spawning area along the eastern coast of mainland China and eastern Japan can reach the spawning area even if swimming slowly and near the surface within 3 to 5 months by exploiting a strong southward-flowing current situated north of the spawning area. Our simulations show that few starting locations give eels the option to swim slowly near the surface if they want to reach the spawning ground within 5 months, and if they do then they must follow along contorted path to avoid strong currents. An increase in speed from 0.1 to 0.2 m/s allows eels to swim against stronger currents, significantly increasing the number of eels that can complete their migration within 5 months and expanding their range of departure.

Swimming near peak routine speed increases eels’ options for reaching the spawning area even if swimming at the surface (0.4 m/s, Fig. 2(a3); 0.6 m/s, Fig. 2(a4)). Once they outswim the westward NEC, eels can initiate migration from areas between the spawning ground and the islands of Taiwan and the Philippines. At a swimming speed of 0.4 m/s, the estimated migration time is 1 to 2 months for eels starting from the eastern coast of Japan; 2 to 3 months from the eastern coast of Mainland China; 3 to 4 months from the islands of Taiwan and the Philippines. Eels swimming at 0.6 m/s can reach the spawning area within approximately 2 months from all Western Pacific coasts. Swimming at higher speeds allow eels to travel faster and along more direct routes.

Swimming at deeper depth has a similarly positive effect on migration routes as increasing swimming speed. At deeper depths, the NEC and the KC weaken and migration paths straighten (note the increasingly radial pattern of optimal paths with increasing depth in Fig. 2; compare panels in the same row). At a depth of 600 m, migration paths radiate out from the spawning area in almost straight times and migration time becomes proportional to the geographic distance between the departure point and the spawning area (Fig. 2(c1-4)). Our model suggests that eels can minimize migration time by swimming in deeper waters to avoid adverse surface currents. However, it is important to note that the comparison between Fig. 2 (a1), (b1), and (c1) shows that at extremely low swimming speeds (0.1 m/s), eels cannot complete their migration solely relying on their own speed without effectively utilizing favorable currents as described in the Zermelo’s solution. This indicates the existence of a speed range where deep swimming is insufficient for migration, but successful migration can be achieved by fully leveraging surface currents.

The Tortuosity Index (${\eta }_{tort}$) provides a quantitative measure of the complexity of an optimal migration path:

$${\eta }_{tort}={S}_{traj}/{S}_{sphr}$$

It is defined as the ratio of the length of the optimal migration path (${S}_{traj}$) to the shortest spherical distance (${S}_{sphr}$) between the departure point and the destination and reaches a minimum value of 1 for straight lines. In eels, both the magnitude and variation of ${\eta }_{tort}$ decrease markedly with increasing swimming speed and depth, with the median ${\eta }_{tort}$ approaching unity at the highest swimming speed and depth examined in this study (Fig. 3(a1-a2)).

Sea currents cause path length ${S}_{traj}$ to differ from swimming distance ${S}_{swim}$, calculated using the formula:

$${S}_{swim}=U\cdot T$$

with *U* the eels’ swimming speed relative to the water, and *T* the migration time. Within this study’s parameter space, increasing swimming speed caused a negligible influence in swimming distance (the relevance value is approximately zero in Fig. 3(b1)), while increasing swimming depth causes a moderate decrease (a moderate, negative relevance value in Fig. 3(b2)). In summary, while swimming deeper and faster are both effective strategies to reach the spawning along a shorter migration trajectory, swimming deeper is more effective because it also minimizes distance swum.

### Trade-off between time and energy

Animals are constrained by the energetic and time demands of migrations. Eel can reduce migration time most effectively by increasing swimming speed (Fig. 3(c1)), followed by reducing migration distance by swimming at greater swimming depth (Fig. 3(c2)), and in just a few cases by increasing migration speed by exploiting favorable currents.

Animals that reduce migration time by increasing speed face the dilemma that going faster requires more energy. They can supply that energy either by interrupting their migration to refuel, which increases migration time, or by carrying more fuel, which reduces fuel efficiency by increasing body weight. Eels opted for the second strategy and do not feed at all while migrating and now face another tradeoff—swimming faster reduces migration time while increasing energetic cost. Whether the decrease in migration time outweighs the increase in energetic cost depends on the relation between swimming speed and energetic cost. In this study, we contrast two different relations, one based on hydrodynamic principles and one based on metabolic measurements.

In the first measurement, we estimate the relation between migration cost and swimming speed based on hydrodynamic principles, which predicts that cost expressed as power (rate of energy use) is proportional to *U*^3^. We express power as fat consumption rate, using data from adult European eels^42^ to estimate energetic cost as follows:

1 $${P}_{total}={P}_{0} + \frac{1}{{\eta }_{phy}}{P}_{hydro}$$

where, ${P}_{total}$ represents total power, ${P}_{0}$ is the basal metabolic power at rest, and ${P}_{hydro}$ is the mechanical power required to overcome fluid resistance. For a fish with a body length of meter-level, ${P}_{hydro}$ is approximately proportional to the cube of swimming speed. ${\eta }_{phy}$ is the energy conversion efficiency from physiological to hydrodynamic power. If ${\eta }_{phy}$ does not change drastically with swimming speed, then ${P}_{total}$ is primarily determined by the cubic relationship with swimming speed. Based on known energy consumption rates at speeds of 0 and 0.5 m/s in experimental observations^42^, we obtain the following equation for eels:

2 $${P}_{fat}=0.02022 + 0.2072 {U}^{3}$$

where ${P}_{fat}$ is the fat consumption rate (measured in g fat per day) and *U* is swimming speed (measured in m/s).

In the second measurement, we estimate the relation between cost and swimming speed based on oxygen consumption measurements, again using data from European eel^70^. This approach circumvents the assumption that ${\eta }_{phy}$ does depend on swimming speed. Yet new caveats arise from methodological limitations of metabolic measurements on fish, which are confounded by the fish’s behavior. Cost and speed appear directly proportionality (coefficient close to unity) for so-called complex swimming behaviors when fish frequently change speed and heading, yet cost and speed approach the cubed relation predicted from hydrodynamic principles during so-called forced swimming, when the fish maintains the same heading^71–75^. The experimentally observed value for eels is close to unity^70^, yielding the following equation:

3 $${P}_{fat}=0.02022 + 0.0518 U$$

To calculate the total amount of fat consumed to reach the spawning ground (${C}_{fat}$), we integrate each of the two equations along the predicted migration paths and obtain markedly different outcomes for the two measurements Fig. 3d.

The predictions based on hydrodynamic principles follow a weak U-shaped trend in the speed-specific median fuel cost (Fig. 3 (d1)), with the minimum at a speed of 0.4 m/s. Total energy varies widely around each median, and the range increases considerably with swimming speed, reaching values exceeding 150 g at 0.6 m/s. In contrast, the metabolic measurement yields a trend with a negative slope — total fuel demand gradually decreases as swimming speed increases. Whereas the hydrodynamic measurement predicts that eels should swim at a moderate speed for optimal migration cost or at a high speed to reduce migration time, the metabolic measurement predicts that eels should swim at their maximum sustainable speed to simultaneously reduce migration time and cost. Unlike speed, swimming depth has a relatively weak effect on energetic cost, even in the hydrodynamic measurement (Fig. 3 (d2)). At low depth, eels benefit from ‘going with the flow’, at greater depth they benefit from straighter paths, resulting in similar costs at all depths.

### Effects of geographic location and season

We examined optimal migration paths for two seasons (winter, and summer) and six regions: North Japan, South Japan, North Chinese Mainland, South Chinese Mainland, Taiwan Island, and the Philippine Islands (Supplementary Information, §S1). The geographic location of the starting point has a strong effect on the lengths of the optimal migration paths ${S}_{traj}$, but only a weak effect on optimal migration time and energetic cost of migration.

We examined the effect of the season by using two monthly average dataset for the West Pacific Ocean from January and July 2020, respectively (Supplementary Information, §S2). Currents are noticeably stronger in summer, leading optimal migration paths to diverge considerably between the two seasonal scenarios and making it impossible for eels to reach the spawning grounds from starting locations along the Western Pacific coast that allow successful migration in the winter season. In general, migration appears to be more challenging in the summer than in the winter.

## Discussion

### What we learn from these benchmark results

In this study, we examined eel migration routes to their spawning site using Zermelo’s theoretical model to predict how ocean currents affect migration. By modeling a range of scenarios, we examined how behavior (swimming speed and depth) and environment (geographic, seasonal, and depth variation of ocean currents) affect migration outcomes. Our results benchmark the amounts of time and energy that eels must invest for a range of real-world scenarios. This study employs two measurements to analyze the energy consumption of eel migration, resulting in significant differences. The hydrodynamic measurement predicts that eels should swim at a speed that is close to the optimal cost of transport during migrations, while the metabolic measurement suggests that eels should swim at their maximum sustainable speed to reduce both migration time and cost. Determining which measurement more accurately reflects the actual energy consumption of eels requires further physiological quantitative experiments.

Silver eels do not refuel during their migrations, and their initial fat reserves range from 10 to 28% with a mean of 20% of total body weight^76^. Our simulations show that eels can complete migrations with plenty of fuel to spare, consistent with experimental findings^42^. The results indicate that, regardless of which energy-consuming measurements (Eq. 2 or Eq. 3) are used, the fat required for migration is sufficient for a virtual eel with 20% body fat when the speed is below 0.6m/s. Tracking data of eels suggest that eels’ migration speed is below 0.5 L/s (body-length per second)^57^, with confirms our model prediction that eel swim slowly to lower the energetic cost of migration at the expense of a longer migration time. Our predicted migration routes from starting points on the Eastern coast of Japan also agree with observations from tagging data^57^ and align well with the predicted routes that assumed that eels exploit the northern Kuroshio^57,76,77^.

Since swimming energy consumption is roughly proportional to the cube of the speed, increasing the speed beyond the range studied here will ultimately lead to a significant rise in energy expenditure, making it unsustainable to maintain the metabolic measurement (Eq. 3). Consequently, regardless of the energy-consuming measurement used, there will be a trade-off between swimming speed and energy consumption. Additionally, field observations of Japanese eels have recorded many individuals around 50 cm in length (especially males). According to our analysis (Supplementary Information, §S3), reduced body length significantly increases swimming energy expenditure from a fluid dynamics perspective. This suggests that smaller individuals swimming at higher speeds are likely to face a real risk of depleting their energy reserves.

### Predicting probable strategies for eels

By simulating outcomes for a range of input values, Zermelo’s deterministic model can provide parameter space maps to assess the robustness and probability of particular model predictions. We examined how behavioral choices affect migration outcomes. In this manner, we can discover which migration strategies emerge as the most advantageous based on how much they affect favorable model outcomes (minimal migration time, distance, and energy).

**Fig. 4.**
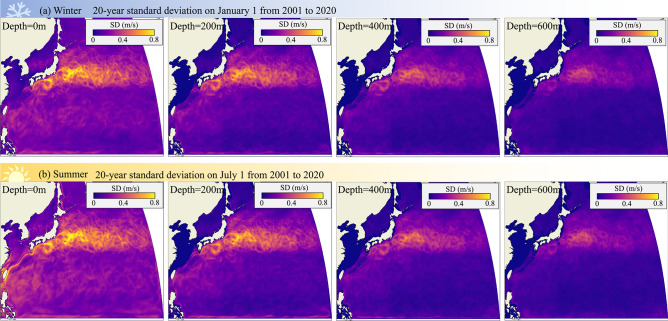
20-year Standard deviation of ocean current vector field at various depths. (**a**) on each January 1 from 2001 to 2020, and (**b**) on each July 1 from 2001 to 2020.

**Fig. 5:**
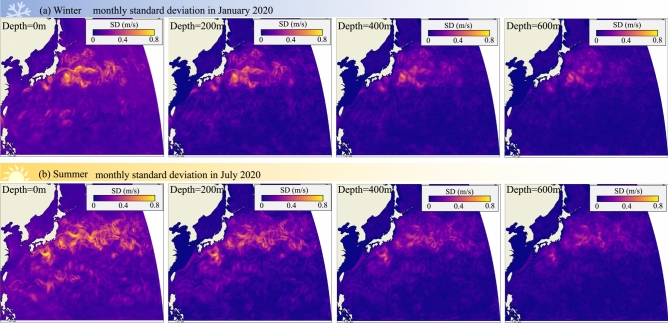
Monthly Standard deviation of ocean current vector field at various depths. (**a**) in January, 2020, and (**b**) in July, 2020.

We found that three of the four migration outcomes are profoundly affected by swimming speed (strongly affected: migration time, energy, route tortuosity; weakly affected: swimming distance) (Fig. 3). Swimming speed has a stronger effect than swimming depth due to the non-linear relation between swimming speed and energy, forcing eels to use relatively low swimming speeds if they are to have enough fat reserves left for reproduction. Furthermore, both swimming speed and swimming depth affect not only the predicted median value of a particular migration outcome, but sometimes also its variability—at low swimming speeds and depth, the predicted optimal migration paths have a complex shape, expressed by the Tortuosity Index (${\eta }_{tort}$) (Fig. 3(a1, a2)). At extremely low swimming speeds, the utilization of ocean currents may be crucial. As depicted in Fig. 2 (a1) and (c1), even though migration paths become more convoluted at lower depths and swimming speeds, it is still possible to complete the migration within the 5-month timeframe established in the study. Conversely, without the influence of currents at a depth of 600 m, eels are unlikely to migrate successfully. While eels swimming near the surface can occasionally take advantage of favorable flow corridors to reduce energy consumption, in most cases, variable currents not only result in longer and more convoluted migration routes but also make them less predictable. This necessitates migrating eels to constantly adjust their migratory path to avoid unfavorable currents. In addition to geographic, seasonal, and depth variation, ocean currents display considerable variability as evident in the large standard deviations for a given depth bracket (Figs. 4 and 5). Such variability implies that eels’ optimal migration paths are not particularly robust at low swimming speeds and depths, especially for routes that cross major ocean currents such as the Kuroshio Current. According to our results, eels should favor depths below 500 m to avoid strong, fluctuating ocean currents. Increasing swimming speed and depth straightens the optimal migration path (${\eta }_{tort}$ closer to 1), allowing eels to swim effectively in a straight line along the predicted optimal migration path as long as they know the exact location of their destination through information such as magnetic field^78,79^. American eels migrating to the Sargasso Sea have been observed to swim straight towards the spawning area once they enter deeper waters^80^, supporting the predictions of our study. In reality, although eels may not have full information about ocean flows as we assumed in this study, they likely possess some innate understanding of the high variability of these flows and the associated risks of migration failure, allowing them to avoid strong ocean currents altogether.

Our model predicts that the most plausible migration strategy involves adopting speeds not far exceeding 0.4–0.6 L/s (based on the hydrodynamic measurement), swimming at depths greater than 200 m, and migrating along straight routes to the spawning area. The swimming depth chosen by real eels is generally in line with this prediction. Evidence from satellite tagging studies indicates that tropical and temperate eel species exhibit pronounced diel vertical depth change during their migration, from between 150 and 300 m nighttime depths to 600–800 m during the day^81^. Both the daytime and nighttime depths are sufficient to avoid surface currents and their seasonal variations, which also suggests that real eels do not attempt to exploit the possible beneficial effects of surface currents, possibly because they are highly variable and unpredictable.

### Implications of model assumptions

Zermelo’s model makes several simplifying assumptions, two of which—purely horizontal migrations and perfect knowledge of ocean currents—are not realistic. In the following, we examine both assumptions and discuss their implications for our predicted optimal migration routes.

#### Assumption of perfect knowledge

One assumption of Zermelo’s model is that migrants have complete knowledge of the ocean’s flow field, whereas in reality, perfect knowledge of ocean currents is not possible. Ocean currents fluctuate on short- and long-term time scales, making navigating those currents a stochastic process where migrating animals need to weigh the potential benefits of exploiting beneficial currents for energy- and time savings against the likelihood of such beneficial currents materializing^13^. We quantified the variability of ocean currents on a short- and long-term time scale by calculating the standard deviation of the mean flow after averaging flows over 20 years of the same calendar day (winter season: January 1st 2001 to 2020; summer season: July 1st 2001 to 2020) (Fig. 4), and after averaging flows over 1 month (winter season: January 2020; summer season: July 2020) (Fig. 5). On both time scales, ocean currents are most variable near the surface and during the summer season. The largest variability occurs within the Kuroshio current, where the standard deviation has the same order of magnitude as the average current speeds; the flow is less variable outside the Kuroshio current, with the standard deviation dropping to one order of magnitude lower than average current speed. These low fluctuations suggest that eels might not encounter highly variable flows outside the Kuroshio current, and can blunt the impact of the Kuroshio current by swimming at greater depths and by migrating during the winter season.

#### Assumption of purely horizontal migration

**Fig. 6:**
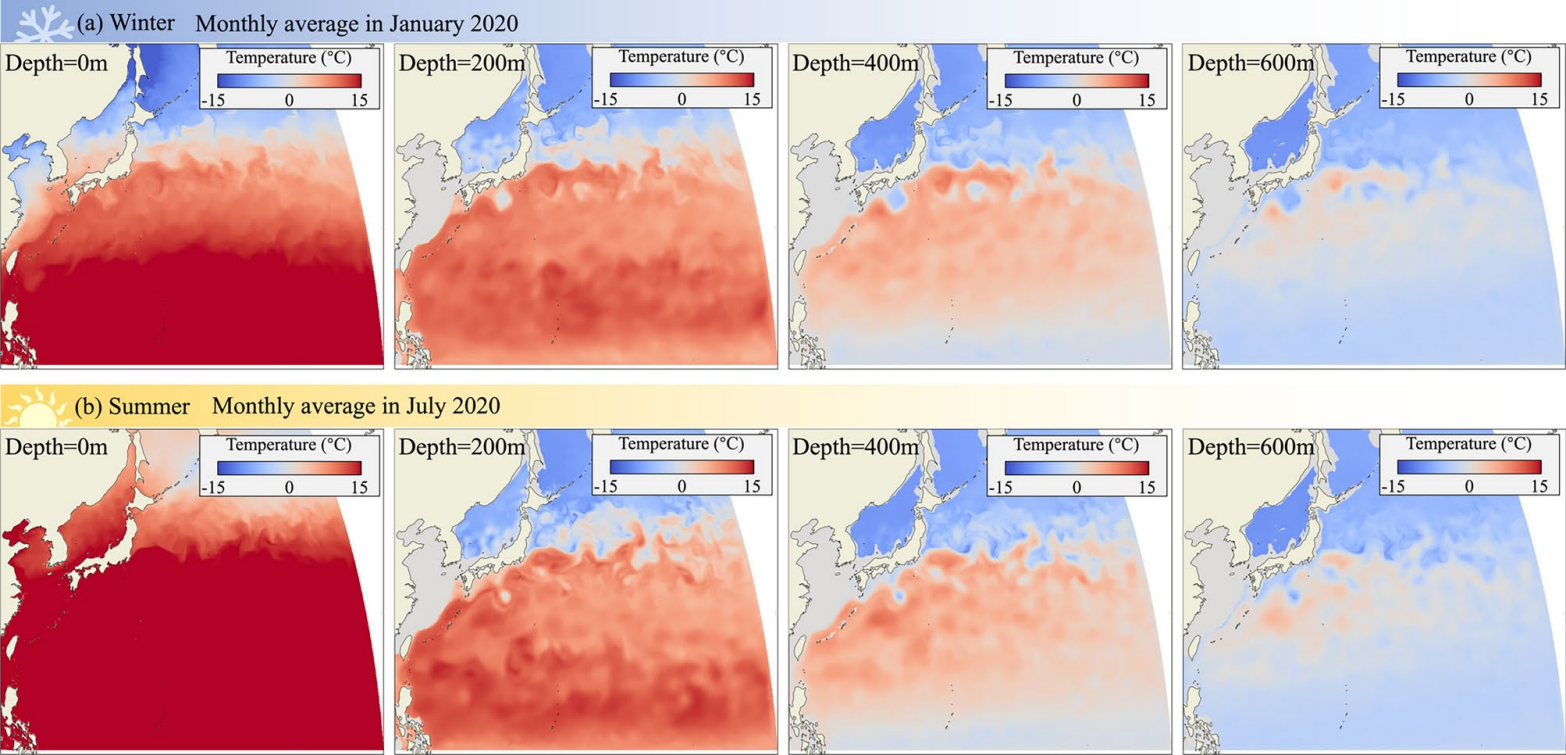
Water temperature at various depth. (**a**) Monthly average in January, 2020, and (**b**) monthly average in July 2020.

Our model assumes purely horizontal migration, yet eels perform diel vertical migrations during their migration to the spawning grounds. While these diel vertical migrations have a negligible effect on total distance travelled and hence the predicted travel distance and duration, they might affect optimal migration routes because water temperature and flow conditions change substantially with depth. To assess the effects of diel vertical migrations on the predicted routes, we calculated migration routes for a range of depths (Fig. 2), and also characterized the flow and temperature conditions at a range of depths (Figs. 4, 5, 6) for both the winter and summer seasons.

Based on ocean current conditions, our model predicts that eels should favor greater depths to avoid strong, highly variable surface currents. Yet water temperature also changes with depth. Our characterization of average temperatures at a range of depths shows the expected general trends of water temperatures decreasing with increasing latitude and depth (Fig. 6). Temperature has a profound effect on muscle contraction physiology, including energetics, and for ectothermic fish that means lower water temperatures cause lower peak muscle power and peak mechanical efficiency at lower muscle contraction speeds^82–85^. This means that in Fig. 3(c2), energy consumption at greater depths may significantly exceed the predictions of the energy model used in this study. It also implies that in low-temperature environments at greater depths, high-speed swimming, which requires high peak muscle power, may not be achievable. Previous studies of eel diel migrations suggested that eels appear to avoid water temperatures below 5 °C^57^. Our temperature maps show that such a threshold of 5 °C would limit eels’ geographic and depth range, especially during the winter season. For example, eels face average temperatures below 5 °C at depths of 600 m in most locations that we mapped, irrespective of season. Our temperature maps suggest that eels face a tradeoff between choosing favorable flow conditions and favorable water temperatures: Ocean currents at the surface are fast and fluctuate considerably, yet water temperatures are mild whereas at depths of 400 m and below, ocean currents are considerably weaker and less variable, but water temperatures are near and often below 5 °C. Observations of eels’ diel migrations show that eels favor warmer surface waters at night and retreat to cooler, deeper waters during the day, possibly to avoid predation^57^.

## Materials and methods

### Characteristics of the virtual eel

The virtual eel in this model is conceptualized as a particle that moves at a constant self-speed relative to the surrounding water, and at a constant depth. We have configured the body metrics (body length = 1 m and body mass = 2 kg) of the virtual eels to align with the data from the study by Van Ginneken and Van den Thillart on European eels^42^, enabling direct comparisons of swimming capabilities and fat consumption with experimental data. These settings also largely conform to the physical data of Japanese eels^69^.

### Mathematical formulation of the eel’s migration

In a horizontal x–y plane, where the flow distribution is given by a vector field as a function of position and time $(u\left(x,y,t\right),v(x,y,t))$, the eel’s ground velocity is the vector sum of the flow velocity and the eel’s swimming velocity relative to the surrounding flow:

4 $$\begin{aligned}\frac{dx}{dt}&=u\left(x,y,t\right)+V\text{cos}\theta \\ \frac{dy}{dt} &=v\left(x,y,t\right)+V\text{sin}\theta \end{aligned}$$

Here, $u$ and $v$ represent the flow speed relative to the ground, *V* is the constant swimming speed of the eel, and $\theta$ is the eel’s heading angle, defined anticlockwise from the x-axis. According to the classical Zermelo’s solution^50^, the optimal headings minimizing the migration time can be expressed as:

5 $$\frac{d\theta }{dt}=\frac{\partial v}{\partial x}{\text{sin}}^{2}\theta +\left(\frac{\partial u}{\partial x}-\frac{\partial v}{\partial y}\right)\text{sin}\theta \text{cos}\theta -\frac{\partial u}{\partial y}{\text{cos}}^{2}\theta$$

For this study, we need to modify those equations to take into account Earth’s curvature. This modification is necessary because the long-distance migration of eels covers a wide range of longitudes and latitudes (10.5–62°N, 108–180°E). Thus, Eqs. (4) and (5) are reformulated for a spherical surface^51,86^. We use the Zermelo’s solution on a sphere derived by McLaren et al.^30^:

6 $$\begin{aligned}\frac{\text{d}\alpha }{\text{d}t} & =\frac{u\left(\alpha ,\beta ,t\right)+V\text{sin}\gamma }{R\text{cos}\beta } \\ \frac{\text{d}\beta }{\text{d}t} & =\frac{v\left(\alpha ,\beta ,t\right)+V\text{cos}\gamma }{R}\end{aligned}$$

Here, $\alpha$ and $\beta$ are the eel’s longitude and latitude, $u$ and $v$ are the eastward and northward flow velocities, $R$ is the Earth’s radius, $\gamma$ is the heading angle clockwise from the geographic north direction, and $V$ is the eel’s velocity relative to the surrounding water.

Zermelo’s solution on the sphere becomes^30^:

7 $$\frac{\text{d}\gamma }{\text{d}t}= - \left(\frac{1}{\text{cos}\beta }\cdot \frac{\partial u}{\partial \alpha }-\frac{\partial v}{\partial \beta }\right)\text{cos}\gamma \text{sin}\gamma +\frac{\partial u}{\partial \beta }{\text{sin}}^{2}\gamma -\frac{\partial v}{\partial \alpha }\cdot \frac{{\text{cos}}^{2}\gamma }{\text{cos}\beta }+ \frac{\text{tan}\beta \text{sin}\gamma }{R}\left(V+u\text{sin}\gamma +v\text{cos}\gamma \right)$$

Here, the last term accounts for the Earth’s curvature^51^; the vertical motion required to maintain altitude over the spherical Earth is neglected^86^; the Coriolis effect is insignificant and neglected^30^.

Zermelo’s solution reduces the problem of solving optimal headings at every potential point to an initial-heading problem. For arbitrary initial heading angles ${\gamma }_{o}$, the heading angle is numerically calculated as:

8 $$\gamma =\int \dot{\gamma }\text{d}t+{\gamma }_{o}$$

where $\dot{\gamma }$ is numerically solved by Eq. (4). Based on Eq. (5), a specific ${\gamma }_{o}$ may specify a trajectory of virtual eel migration.

9 $$\mathbf{S}=\int V\cdot \overrightarrow{{\mathbf{N}}_{\gamma }}\text{d}t$$

In this equation, **S** represents the trajectory of eel migration, and $\overrightarrow{{\mathbf{N}}_{\gamma }}$ is a unit vector with heading angle *γ*.

The optimal initial heading angle ${\gamma }_{o}$ that makes the trajectory of virtual eel migration pass through the destination point of the migration route will be numerically solved. Once the optimal initial heading angle is determined, the optimal migration trajectory that minimizes migration time while travelling at a specific migratory speed is also resolved.

### Numerical strategy

Determining the optimal initial heading angle ${\gamma }_{o}$ by solving Zermelo’s equations numerically requires multiple rounds of simulations, and sometimes those solutions do not yield an optimum but merely a local extremum. To reduce the number of simulations rounds required to find optima, we employed a reverse-time numerical strategy where all equations are solved in a reverse-time system with the reverse-speed ocean current data (e.g., a flow of 1 m/s NE is transformed to 1 m/s SW, or equivalently, − 1 m/s NE). In essence, this approach finds the optimal initial heading by simulating the eel as traveling from all possible destination points to the known start point through an ocean in which the direction of the currents are also reversed. For each possible destination point, we calculated multiple possible trajectories radiating out in all possible directions by varying the initial heading angle ${\gamma }_{o}$ in very small angular steps (${\Delta \gamma }_{o}=0.5^\circ$). Each trajectory that reached the start point was then transformed back to the normal time system, and such a trajectory represents an optimal migration trajectory from an arbitrary point on that trajectory to the destination point. In this way, we found optimal migration trajectories from any starting point to the destination for a specific arrival time with just one round of simulations that scan through possible values of ${\gamma }_{o}$. This reverse-time solution strategy not only significantly enhanced accuracy, but also allowed us to draw iso-migration-time maps and, ultimately, iso-energy-consumption maps. We conducted simulations at a range of observed sustainable swimming speeds^70^ and observed swimming depths during diel vertical migrations^56–58^ to examine their effects on optimal trajectory.

The center of the spawning area for Japanese eels, as reported by Tsukamoto^38^, is set at 15° N, 140° E. Since it has also been reported that the spawning area is relatively wide, we set 15 goal points evenly distributed within the 14–16° N, 138–142° E area for this study.

When eels encounter the continental shelf during simulation, we assume they move closely along the seabed, entering the boundary layer of it, where the current speed can be assumed as zero. The simulation continues in this zero-intensity flow field (for specific explanations, please refer to Supplementary Information, §S4).

The simulation solver was programmed and executed in the Python Development Environment Spyder IDE 5.3.3.

### Ocean flow database and simulation region

To solve Eq. (4) numerically, we required ocean flow data as a function of longitude, latitude, and time. The ocean data used in this study were obtained from the Japan Coastal Ocean Predictability Experiment 2’s (JCOPE2M^65^) ocean circulation forecast system. The JCOPE2M model encompasses the western North Pacific Ocean (10.5–62°N, 108–180°E), with a horizontal resolution of 1/12° (approximately 8–9 km) and 46 vertical layers (reaching a maximum depth of 1500 m). The JCOPE2M is updated daily and covers the period from January 1993 to the present. Further details of the ocean flow model and database are provided by Miyazawa et al.^65^).

To assess the stochastic properties of the flow, we also computed the variability of the flow expressed in standard deviations from the monthly average calculated from all flow values for 1 month (January 2020 and July 2020) as well as calculated from the yearly average, that is values recorded on the same calendar day over 20 years (January 1 or July 1 between 2001 and 2020). To assess the possible effect of water temperature, we calculated the average temperature for the months of January and July 2020 at four depths (0, 200, 400, 600 m). By characterizing how temperature and the variability in flow speed change with depth, we assessed the possible effects of the eels’ diel vertical migrations on the migratory trajectories predicted by our model. To assess the effect of season, we modeled migrations in winter and summer, choosing January as the focal month for the winter migration based on observed times for migration onset^87^, and July to be exactly 6 months later.

## Supplementary Information

Supplementary Figure 1.

Supplementary Information.

## Data Availability

The datasets for Japan Coastal Ocean Predictability Experiment 2’s (JCOPE2M) is accessible on the website of JCOPE (https://www.jamstec.go.jp/jcope/htdocs/e/home.html).

## Code availability

The code is accessible on Open Science Framework repository (10.17605/OSF.IO/ZJ8GD).
