# Supplementary material for: The calculated voyage: benchmarking optimal strategies and consumptions in the Japanese eel’s spawning migration
Source: Sci Rep. 2024 Oct 31;14:26024. doi: 10.1038/s41598-024-74979-0 (PMC11528122; doi:10.1038/s41598-024-74979-0)
Supplement: Supplementary file 2 — Supplementary Information. [file 41598_2024_74979_MOESM2_ESM.pdf]

Supplementary Information

The Calculated Voyage: Benchmarking Optimal Strategies and Consumptions in the Japanese Eel's Spawning Migration

Gen Li[1*], Yu-Lin Chang[2], Yasumasa Miyazawa[2], Ulrike K Müller[3]

[1] Center for Mathematical Science and Advanced Technology, Japan Agency for Marine-Earth Science and Technology, Japan
[2] Application Laboratory, Japan Agency for Marine-Earth Science and Technology, Japan
[3] Department of Biology, California State University, Fresno, USA
*Correspondence: Gen Li (ligen@jamstec.go.jp)

§ S1 Specific Analysis for Eels from Various Geographic Regions

We examine optimal migration paths from six regions: North Japan, South Japan, North Chinese Mainland, South Chinese Mainland, Taiwan Island, and the Philippine Islands (Fig. S1).

Geographic location of the starting point has a strong effect on the lengths of the optimal migration paths S_{traj}, with eels starting from South Chinese Mainland and North Japan facing the longest journeys, typically between 3000 and 4000 km, with some reaching nearly 5000 km (Fig. S2 (a1-a4)). The shortest paths originate on Philippine Islands, with a median S_{traj} falling below 2000 km and, in some instances, plunging to as little as 1500 km. Paths from the remaining regions (South Chinese Mainland, South Japan, Taiwan) are intermediate in length, mostly between 2000 and 3000 km. Median path length and variation in path length within each region decrease as swimming speed and swimming depth increase, with eels originating from southern and northern Japan showing the greatest decrease (Fig. S2 (a1) to (a4)).

Geographic location has a relatively weak effect on optimal migration time (Fig. S2 series (b)). For slow-swimming eels, median migration times hover around 120 to 145 days (Fig. S2 (b1)), yet values range widely within a given geographic region. Eels from North Chinese Mainland take the longest, arriving about 20 days later than those from Philippine Islands and South Japan. Please note that we capped migration time at five months, so results exceeding 150 days were excluded, creating an arbitrary ceiling for migration times. As eels swim faster, median migration time decreases dramatically, as does variation, for all locations. At a speed of 0.6 m/s, the migration time shortens to a range of 40 to 60 days. Eels from North Chinese Mainland still take the longest, arriving about 20 days later than eels from Philippine Islands and South Japan. The (b) series of Fig. S2 indirectly depict the actual distance swum, which is the product of migration time and swimming speed. At the median migration time, distance swum ranges from 2,000 to 3,000 kilometers.

Geographic location has only a weak effect on migration cost (Fig. S2 series (c)). At low speeds, cost is low and similar across geographic location (Fig. S2(c1)), yet at higher swimming speeds migration cost increases markedly and differences between geographic location become more pronounced (Fig. S2(c2)). Notably, eels commencing their journey in North Japan and North Chinese Mainland consume the most fat, in some extreme cases exceeding 100 g.

The six geographic locations show moderate differences in energy cost between low and high swimming depths (Fig. S2(c)). Yet whereas the effects of swimming speed and depth on migration distance (Fig. S2(a)) and time (Fig. S2(b)) are consistent across the six geographic locations, the same is not true for energetic cost: eels from the Philippines see their fat consumption halved as they move from the surface to a depth of 700 m, whereas eels from North Chinese Mainland consume less fat if their swim at the surface.

To better understand the contribution of ocean currents to eel migration, we introduce a measure we call the current ride index e η_{ride}, the current-ride index, defined as the ratio between the actual swimming distance (S_{swim}) and trajectory distance (S_{traj}):

$$\eta_{ride}=S_{swim}/S_{traj}$$

An η_{ride} value smaller than unity implies that the eel is riding the current to reduce its swimming distance while travelling along the optimal migration path, versa value larger than unity indicates that eels swim against the current. According to Fig. S2(d1)-(d4), under low-speed conditions (Fig. S2(d1)), eels from all regions except the Philippine Islands have a median η_{ride} of less than 1, suggesting that eels use currents along their path to reduce the actual swimming distance. Eels starting their journey from the southern and northern parts of Japan and North Chinese Mainland benefit the most from ocean currents. Some eels from South Japan achieve an η_{ride} of 0.5 when completing their optimal migration path, indicating that their actual swimming distance is only half of their trajectory distance. As swimming speed increases to 0.6 m/s (Fig. S2(d2)), η_{ride} generally trends towards 1, suggesting that the benefits gained from ocean currents decrease as speed increases.

For all six geographic locations, swimming slowly and near the surface causes migration paths to deviate further from a straight line than when swimming fast or at greater depth: the tortuosity index (η_{tort}) is considerably larger than unity for low swimming speeds and depth, with eels commencing their migration in North and South Japan facing the highest tortuosity and highest variation in tortuosity (Fig. S2(e1),(e2)). Median tortuosity and variation in tortuosity are consistently close to unity at the higher swimming speeds and greater swimming depths (Fig. S2(e3),(e4)) across all six locations.

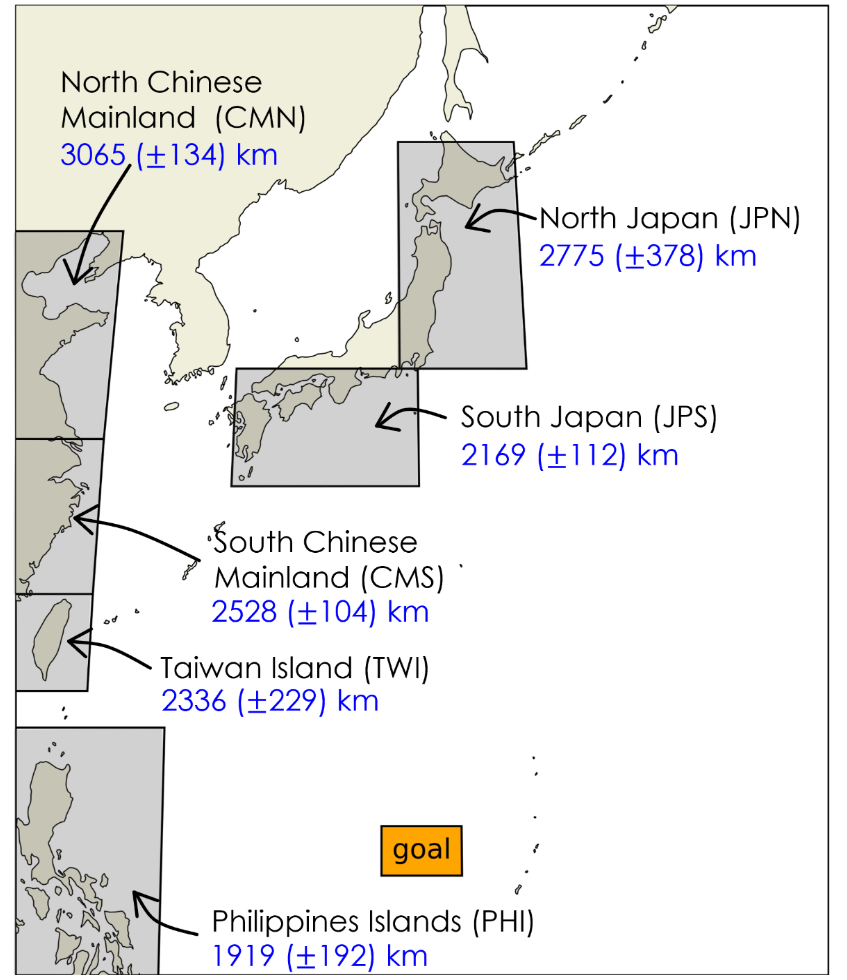

Figure S1. 6 Habitant Zones defined in our analysis.

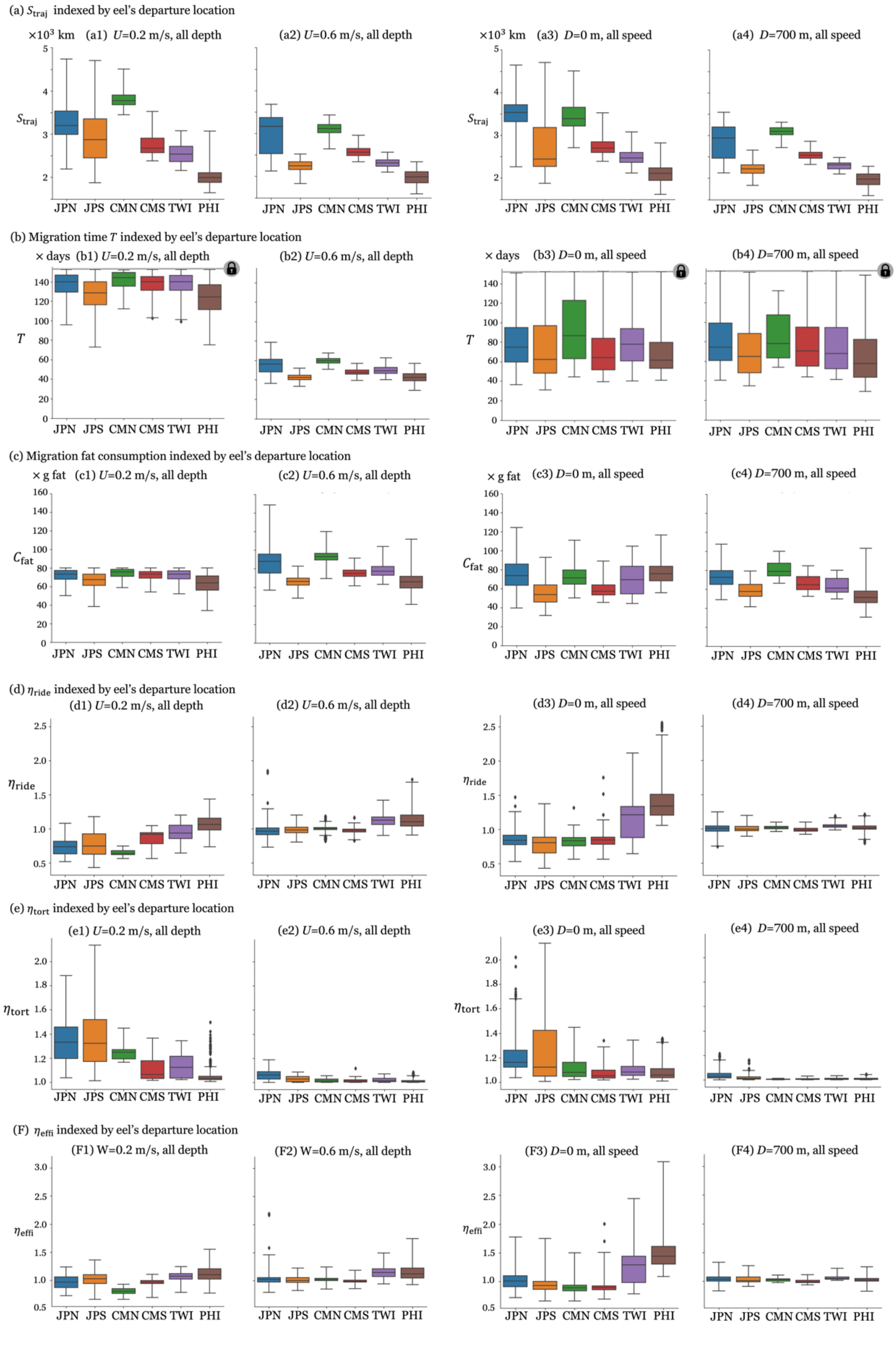

Figure S2. Specific analysis for eels from 6 various habitants: North Japan (JPN), South Japan (JPS), North Chinese Mainland (CMN), South Chinese Mainland (CMS), Taiwan Island (TWI), and the Philippine Islands (PHI).

§S2 Seasonal effect: simulations in summer scenario

The flow field data used for the Summer Scenario were obtained from the monthly average dataset for the West Pacific Ocean from July 2020. Compared with the Winter Scenario, the currents in the Summer Scenario are noticeably stronger. The velocities of both the Kuroshio Current (KC) and North Equatorial Current (NEC) increase (Liu & Zhou, 2020; Wei et al., 2015), with more vigorous circulation patterns present throughout the entire region. The optimal migration paths computed for the Summer Scenario noticeably diverge from those generated in the Winter Scenario. Eels swimming at the lowest speed (0.1 m/s) modelled in our simulations are unable to reach the spawning ground from any location along the Western Pacific coast within 150 days, the maximum threshold for migration time used in our simulation, irrespective of swimming depth (Fig. S3(a1)). Increasing swimming speed to 0.2 m/s allows eels commencing their migration from the Philippine Islands and Japanese Islands to reach the spawning ground within the maximum time of 5 months allowed by our simulation (Fig. S3(a2)). Optimal migration paths originating from the eastern coast of China and Taiwan Island are noticeably distorted and scattered, taking detours around many empty areas. Eels originating from these coastal regions must avoid powerful currents and make use of favorable flows, a strategy that is not only demanding and complex but also requires eels to have a thorough knowledge of the dynamics of the flow field.

Similar to the Winter Scenario, increasing swimming speed and depth result in less contorted optimal migrations paths, simplifying the navigation process for eels through the flow field (Fig. S3). Optimal migration paths become increasingly less contorted as swimming depth increases (see Fig. S3(b1-b4)) and approach the pattern of straight paths radiating out from the spawning area (Fig. S3 (c1-c4)) observed in the Winter Scenario (Fig. 2) at a depth of 600 m or deeper (Fig. S3 (c1-c4)).

In general, seasonal differences in optimal migration paths are most evident for eels migrating near the surface at slower speeds (compare Fig. S3 (a1- a2) with Fig. S3 (a1- a2)). However, when eels swim at significant depths exceeding 200 m, the seasonal impacts dramatically decrease. The results suggest that migrations in the Summer Scenario are more challenging when swimming near the surface due to stronger flows unfavorable for migration, but the overall trends are the same as in the Winter Scenario, and Summer and Winter Scenario become similar at depths greater than 200 m.

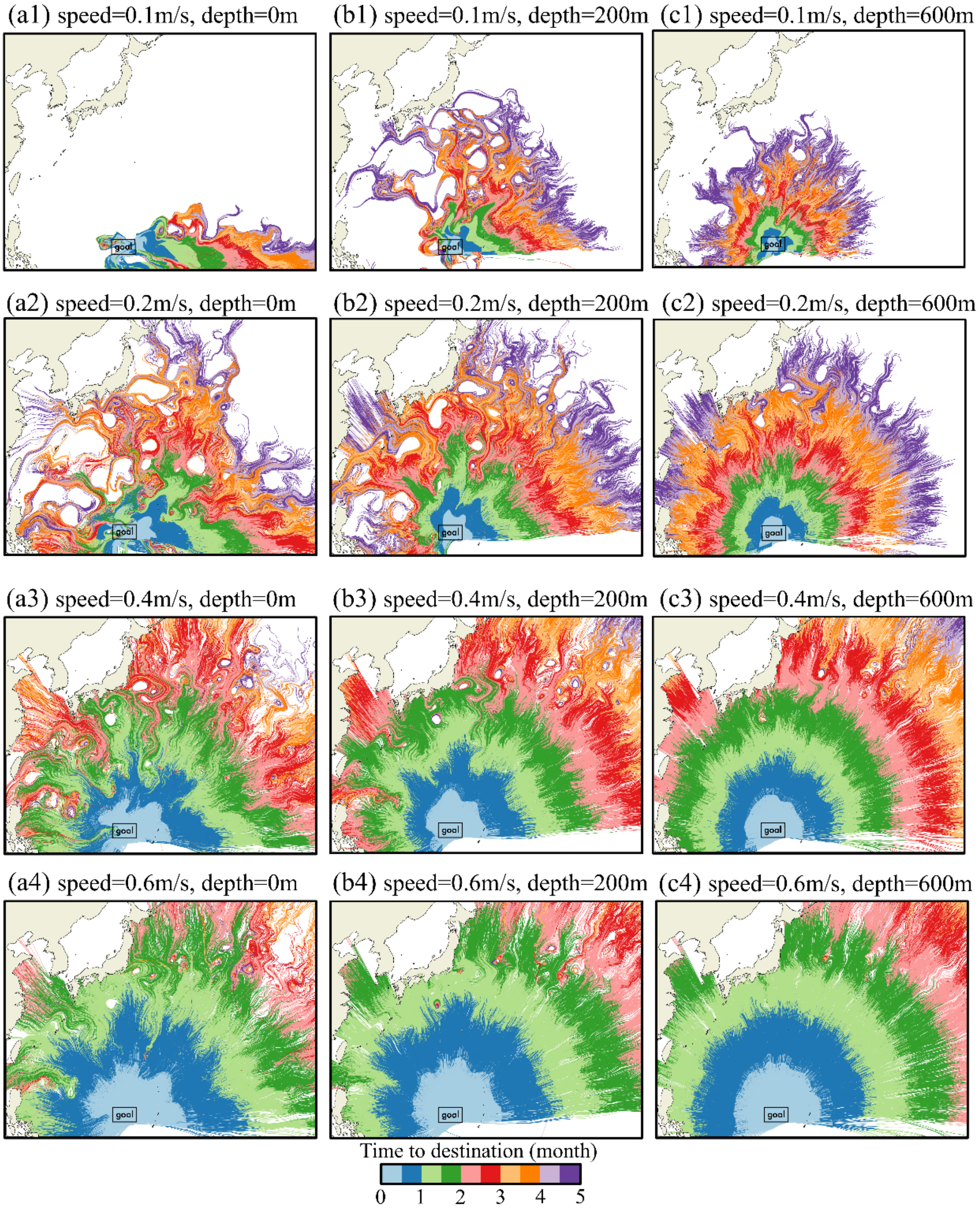

Figure S3. Optimal migration path in the Summer Scenario at Various Depths. (a1-a4) depict optimal migration paths at the surface (Depth=0 m); (b1-b4) illustrate optimal migration paths at a moderate depth (Depth=200 m); (c1-c4) present optimal migration paths in the deep sea (Depth=600 m).

§S3 Impact of reduced body length on swimming energy expenditure

It is essential to recognize the impact of reduced body length on swimming energy expenditure from a fluid dynamics perspective. In this study, the virtual eel is assumed to have a length of 1 meter and a weight of 2 kilograms. These assumed body metrics are consistent with observations of migratory Japanese eels in the ocean, nevertheless, many body length measurement records for Japanese silver eels are usually around 50cm, smaller than our virtual eels. For an eel with a characteristic length of 1m swimming at a speed of 0.5 m/s, its Reynolds number is in the order of 10^6, and even if its body length is halved, the order of magnitude of the Reynolds number does not change significantly. From a dimensionless analysis perspective, in the dimensionless mechanical power of swimming ($P^* = P\rho^{-1}U^{-3}L^{-2}$, where ρ is the fluid density, U is the swimming velocity, L is the body length) does not change significantly. With the dimensional swimming velocity U remaining constant, the dimensional mechanical power P is proportional to L^2, while the eel's mass and its fat storage are approximately proportional to L^3 . Thus, a rough estimate suggests that the mechanical power of a 50 cm long eel at the same speed would be 1/4 of that of a 1m long eel, while its fat storage would only be 1/8. Incorporating these ratios into the results of Fig. 3(d1) in the main text, it can be concluded that a 50 cm long eel would have much tighter fat reserves after completing its migration, and may even use out fat reserve in some extreme conditions, especially when the speed in greater than 0.6m/s under the "hydrodynamic measurement". Therefore, the success rate of eel migration is sensitive to body length, with smaller eels facing significantly increased pressure on their fat reserves during migration.

§ S4 Simulation protocol when eels collide with the continental shelf at a specific depth

In this study, we used a computational system that reverses time and flow field velocity, meaning that the eels start from the spawning area and move towards freshwater habitats. When eels migrate at greater depths, there is a possibility of collision with the continental shelf. In this case, our approach is based on the following assumptions:

(1) When eels encounter the continental shelf when swimming at greater depths with horizontal speed U (Figure S4, Point A), we assume they will move closely along the seabed (Figure S4, Curve A-B).

(2) Due to the friction effects between seawater and the seabed, a boundary layer is generated near the seafloor. Therefore, when eels are moving closely along the seabed, we can assume that the current speed they experience is zero.

(3) Since eels can adjust their buoyancy using organs such as their swim bladder, their horizontal speed remains at the original migration speed while they are moving along the seabed, influenced by a zero-speed current.

(4) Thus, eels will progress along Curve A-B at the horizontal migration speed U. In the simulation solution, this essentially means that after the eels swim horizontally at their original depth and touch the continental shelf, they continue to move horizontally at the original speed (Figure S4, Line A-C), while the equivalent flow field speed after virtually entering the continental shelf is zero.

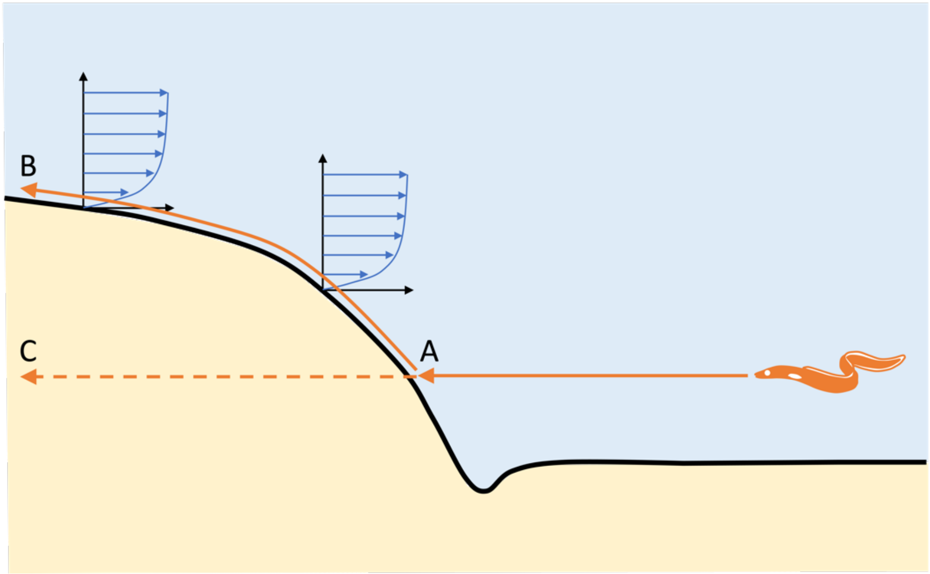

Figure S4. Schematic diagram regarding the simulation protocol for eels colliding with the continental shelf at a specific depth in this study." The two paths, Curve AB and Straight-line AC, are essentially equivalent in the solution.

Through this portocol, we can still derive results under the conditions of (1) not violating the conditions for Zermelo's solution, (2) adhering to the principles of fluid mechanics, and (3) not discarding meaningful data.

§ S5 Additional Statistical Data for Figure 3

In order to gain a better understanding of the data and statistical significance in Figure 3, we conducted a linear regression analysis and presented the analysis results in Table S1.

Table S1. Linear regression analysis for Figure 3

**Location**	**Dep. Variable**	**Indep. Variable**	**Intercept**	**Slope**	**Adjusted R2**	**Slope p-value**	**Dep. Variable's std deviation**
Fig.3 a1	η_tort	U	1.1871	-0.2934	0.170	<0.001	U=0.2: 0.181926<br>U=0.3: 0.099267<br>U=0.4: 0.060898<br>U=0.5: 0.038200<br>U=0.6: 0.026957
Fig.3 a2	η_tort	D	1.2349	-0.0003	0.230	<0.001	D=0.0: 0.199652<br>D=100.0: 0.159531<br>D=200.0: 0.127098<br>D=300.0: 0.103905<br>D=400.0: 0.089966<br>D=500.0: 0.066860<br>D=600.0: 0.057522<br>D=700.0: 0.035334
Fig.3 b1	S_swim	U	2.35	0.793	0.046	<0.001	U=0.2: 0.280642<br>U=0.3: 0.450250<br>U=0.4: 0.486522<br>U=0.5: 0.502652<br>U=0.6: 0.488127
Fig.3 b2	S_swim	D	2.738	0.0069	0.102	<0.001	D=0.0: 0.565452<br>D=100.0: 0.472569<br>D=200.0: 0.466771<br>D=300.0: 0.435801<br>D=400.0: 0.419138<br>D=500.0: 0.427209<br>D=600.0: 0.440893<br>D=700.0: 0.459074
Fig.3 c1	T	U	158.3423	-194.117	0.724	<0.001	U=0.2: 19.011414<br>U=0.3: 20.139194<br>U=0.4: 14.665318<br>U=0.5: 10.820654<br>U=0.6: 8.654753
Fig.3 c2	T	D	79.7157	-0.011	0.007	<0.001	D=0.0: 28.566984<br>D=100.0: 29.756780<br>D=200.0: 29.243090<br>D=300.0: 29.182486<br>D=400.0: 29.098501<br>D=500.0: 30.175666<br>D=600.0: 30.704816<br>D=700.0: 30.341690

(continued in next page)

Table S1 (continued). Linear regression analysis for Figure 3

Fig.3 d1	C_fat	U	hydrodynamic-force-based 55.2422<br><br>hydrodynamic-force-based 83.6262	hydrodynamic-force-based 23.57<br><br>hydrodynamic-force-based -34.3853	hydrodynamic-force-based 0.054<br><br>hydrodynamic-force-based 0.074	hydrodynamic-force-based <0.001<br><br>hydrodynamic-force-based <0.001	hydrodynamic-force-based<br>U=0.2: 9.982179<br>U=0.3: 12.477149<br>U=0.4: 11.784158<br>U=0.5: 11.977166<br>U=0.6: 13.380948<br><br>hydrodynamic-force-based<br>U=0.2: 13.952857<br>U=0.3: 17.284262<br>U=0.4: 14.409555<br>U=0.5: 11.977166<br>U=0.6: 10.655731
Fig.3 d2	C_fat	D	hydrodynamic-force-based 70.9501<br><br>hydrodynamic-force-based 78.0810	hydrodynamic-force-based -0.0161<br><br>hydrodynamic-force-based -00150	hydrodynamic-force-based 0.077<br><br>hydrodynamic-force-based 0.035	hydrodynamic-force-based <0.001<br><br>hydrodynamic-force-based <0.001	hydrodynamic-force-based<br>D=0.0: 15.017173<br>D=100.0: 12.265388<br>D=200.0: 12.224363<br>D=300.0: 11.503586<br>D=400.0: 11.879601<br>D=500.0: 12.555384<br>D=600.0: 12.829366<br>D=700.0: 13.172278<br><br>metabolic-measurement-based<br>D=0.0: 18.144363<br>D=100.0: 18.710219<br>D=200.0: 18.362419<br>D=300.0: 17.914295<br>D=400.0: 17.425075<br>D=500.0: 17.759561<br>D=600.0: 17.992475<br>D=700.0: 18.040526
